# Consumer Perceptions and Sustainability Challenges in Game Meat Production and Marketing: A Comparative Study of Slovakia and the Czech Republic

**DOI:** 10.3390/foods14040653

**Published:** 2025-02-14

**Authors:** Martin Němec, Marcel Riedl, Jaroslav Šálka, Vilém Jarský, Zuzana Dobšinská, Milan Sarvaš, Zuzana Sarvašová, Jozef Bučko, Martina Hustinová

**Affiliations:** 1 Department of Forestry and Wood Economics, Faculty of Forestry and Wood Sciences, Czech University of Life Sciences Prague,165 00 Prague, Czech Republic; riedl@fld.czu.cz (M.R.); jarsky@fld.czu.cz (V.J.); dobsinska@tuzvo.sk (Z.D.); zuzana.sarvasova@nlcsk.org (Z.S.); 2 Department of Forest Economics and Policy, Faculty of Forestry, Technical University in Zvolen, 960 01 Zvolen, Slovakia; salka@tuzvo.sk; 3 Centre for Knowledge Transfer and Forest Pedagogy, National Forest Centre, 960 01 Zvolen, Slovakia; milan.sarvas@nlcsk.org; 4 Science and Research Division, National Forest Centre, 960 01 Zvolen, Slovakia; 5 Institute for Forest Resources and Information, National Forest Centre, 960 01 Zvolen, Slovakia; jozef.bucko@nlcsk.org; 6 Faculty of Agrobiology and Food Resources, Institute of Nutrition and Genomics, Slovak University of Agriculture in Nitra, 949 76 Nitra, Slovakia; priroda@polovnictvo.sk; 7 Slovak Hunting Chamber, 841 03 Bratislava, Slovakia

**Keywords:** game meat production, consumer perceptions, sustainability, Slovakia, Czech Republic, sustainable agri-food systems

## Abstract

Game meat production represents a unique opportunity to align ecological sustainability with the growing consumer demand for sustainable agri-food products. This study focuses on the perspectives of processors and landowners in Slovakia and the Czech Republic, examining their views on market trends, customer behaviours, barriers, and sustainability challenges. Focusing on these key stakeholders, the study highlights their central role as key drivers in shaping and sustaining the game meat value chain. This research combines secondary data analysis and in-depth interviews with key stakeholders to provide a comprehensive understanding of the game meat sector. Findings highlight that, while game meat is valued for its organic and sustainable qualities, barriers such as limited consumer awareness, high costs, and regulatory constraints hinder its market potential. The study reveals the vital role of consumer education, branding, and the development of value-added products in bridging the gap between ecological management and sustainable market growth. Moreover, the research underscores the need for tailored policies to address structural inefficiencies, promote collaboration across the value chain, and enhance accessibility to sustainable game meat products. By aligning production and marketing strategies with consumer preferences, the sector can contribute significantly to sustainable agri-food systems while supporting rural economies and biodiversity conservation. This study provides actionable recommendations for industry stakeholders and policymakers aiming to foster sustainable practices and consumer engagement in the game meat market.

## 1. Introduction

### 1.1. Context and Importance

The production of game meat, which involves obtaining meat from wild or farmed game, is an important non-timber forest product with significant value in various ways. It supports the economic sustainability of rural areas and plays a crucial role in ecological management and conservation efforts. Therefore, this field of study is highly compelling for professionals in forestry and wildlife management [1,2,3,4,5,6]. Economically, game meat production contributes to rural economies by providing income opportunities through hunting tourism, meat processing, and related activities [7,8,9,10,11,12]. This is particularly significant in regions where traditional agriculture may be less viable due to geographical and climatic conditions. The high nutritional value and growing consumer demand for organic and sustainably sourced meat products further enhance the market potential of game meat [3,12,13,14,15].

In Central Europe, particularly Slovakia and the Czech Republic, game meat production holds historical significance and contemporary relevance. This study specifically focuses on the production of game meat derived from wild ungulates, which are prevalent in the forests of Central Europe and respective countries. Ungulates represent a significant portion of the game meat market, and their management is crucial for both ecological balance and the sector’s economic viability. They are an integral part of local biodiversity and cultural heritage [14,16]. Sustainable management practices, including regulated hunting and game farming, are essential for maintaining ecological balance and preventing overpopulation, which could otherwise lead to environmental degradation [17,18].

Despite its importance, research on game meat production in Central Europe has been somewhat fragmented and limited in scope. Studies have often focused on specific aspects, such as hunting quotas, population dynamics, or meat quality, without fully integrating the ecological, economic, and social factors [6,19,20,21]. This study addresses this gap by adopting a comprehensive approach that examines these interconnected dimensions to provide a holistic perspective on the game meat sector. Moreover, there is a notable lack of comparative analyses between countries like Slovakia and the Czech Republic, resulting in an incomplete understanding of regional differences and shared challenges in game meat production. Research in Central Europe has mainly addressed the dynamics of the unique market and challenges of the game meat industry. For example, Němec et al. [22] explored consumer attitudes towards game meat in the Czech Republic, identifying significant factors influencing market segmentation and development. Similarly, Proskina et al. [12] examined the market position of non-traditional agricultural products, including game meat, providing insights into regional consumer preferences and market potential. Global studies on game meat production and marketing have underscored its ecological benefits, economic potential, and contribution to healthy nutrition. For instance, Hoffman et al. [23] highlighted game meat’s appeal due to its low-fat content and high nutritional value. However, challenges such as supply consistency, quality control, and mixed consumer acceptance due to game meat’s distinctive taste and aroma remain largely unresolved [9,24].

Importantly, these challenges create significant opportunities to develop value-added products tailored to diverse consumer preferences [25,26]. Examples of such products include ready-to-cook meals, premium sausages, and processed game meat cuts, which appeal to health-conscious consumers and enhance the industry’s ability to diversify its product portfolio, ensure consistent quality standards, and broaden its market presence. Achieving these outcomes requires a thorough understanding of consumer behaviour. Research by [27,28] demonstrates that attitudes toward animal welfare and hunting play a critical role in shaping purchasing decisions for wild game meat. This highlights the importance of targeted communication strategies and product development that align with consumer values.

Suppliers and processors play a pivotal role in meeting market demands [29,30,31]. Their perspectives on market trends, consumer segments, and competitive dynamics are essential for addressing regulatory barriers, optimising supply chains, and fostering innovation [32,33,34,35,36]. Specifically, this study examines their insights into market accessibility, pricing strategies, and the challenges of adapting to evolving consumer preferences, which are critical to enhancing the value chain’s sustainability and efficiency.

### 1.2. Objectives of the Study

This study conducts a comparative analysis of game meat production, processing, and marketing in Slovakia and the Czech Republic. It addresses sectoral challenges, market trends, and strategic opportunities by examining regulatory barriers, supply chain inefficiencies, and consumer preferences to enhance product availability, quality, and profitability.

The significance of game meat extends beyond its ecological and economic contributions; it also reflects the unique geographical and cultural landscapes in which it is produced. As noted in the previous section, Central Europe—particularly the Czech Republic and Slovakia—offers a compelling context for exploring the interplay between biodiversity, land use, and market dynamics, underscoring the importance of regional characteristics. Understanding these regions’ specific characteristics is critical to framing the challenges and opportunities within the game meat industry.

By examining the perspectives of suppliers and processors, the study unveils essential insights into market dynamics and opportunities for innovation. It integrates field research, stakeholder interviews, and literature reviews to inform the sustainable commercialisation of game meat. These methods underpin policy reforms and industry practices, aligning them with the contextual framework while addressing the following research questions:

- Consumer Behaviour: What influences consumer preferences and purchasing behaviour toward game meat, and how do these differ between Slovakia and the Czech Republic?
- Market Opportunities: What value chain improvements can optimise game meat market growth?
- Production and Regulatory Constraints: What legislative, technical, economic, and natural factors impact game meat production, profitability, competitiveness, and market access, and what reforms are needed to address these challenges?

## 2. Materials and Methods

### 2.1. Secondary Data Collection and Analysis

The initial phase involved systematically gathering relevant documents and reports from online databases, academic libraries, and industry associations, focusing on forestry, hunting, game meat production, and related damages. This phase included an extensive review of academic journals, industry reports, government publications, and market research studies, with particular emphasis on comparative data from Czechia and Slovakia CSO (Czech Statistical Office), MPRS (Ministry of Agriculture and Rural Development of the Slovak Republic), MZe (Ministry of Agriculture CZ), NLC (National Forest Centre), SPK (Slovak Hunting Chamber) and UHUL (The Forest Management Institute Brandýs nad Labem).

The secondary data served as a critical foundation for formulating research questions and designing questionnaires for the field research. This data also enabled a detailed comparison and contextualization of the subsequent field research and interview findings. The results of this analysis are presented through illustrative graphs and comprehensive tables in the Section 3 and are further examined in the Section 4.

### 2.2. In-Depth Interviews

Building on secondary data analysis, qualitative interviews (Appendix A) were conducted between 2022 and 2024 in the Czech Republic and Slovakia to explore the complexities of game meat production and marketing. An in-depth qualitative approach was employed, involving key players in the market, such as forest landowners, game processors, and participants at various levels of the value chain. The semi-structured questionnaire gathered detailed insights into production processes, marketing strategies, market challenges, regulatory issues, and consumer behaviour. This method ensured a comprehensive understanding of the industry’s dynamics, including the perceptions of the market, customers, and marketing plans of these key players, while allowing flexibility to adapt as needed.

The qualitative interview process followed best practices as outlined by Braun et al. [37] for thematic analysis and Kvale et al. [38] for semi-structured interviews. These methods ensure rigorous data collection and analysis, enhancing the study’s reliability and validity. The interview questions were designed following recommendations by Sharp et al. [39], emphasising open-ended and exploratory questioning techniques.

In analyzing the interview transcripts, we employed thematic analysis following the principles established by Braun et al. [37]. This involved initial coding, theme identification, and cross-comparison to ensure comprehensive interpretation.

Participants were selected based on specific criteria to ensure a comprehensive representation of the game meat production industry. Primary producers with significant production volumes and extensive experience were included, along with processors managing a substantial portion of game meat, providing valuable insights into processing challenges and quality control. Additionally, representatives from forestry and hunting regulatory bodies and forest landowners were incorporated to provide a well-rounded perspective on regulatory and land management issues. The selection criteria were therefore the following: (1) active involvement in the game meat value chain as forest land owners, hunters and processors, or policymakers; (2) representativeness to the relevant game meat value chain within Slovakia or the Czech Republic (volume and experience); (3) a minimum of two years of experience in the sector; and (4) willingness to participate in the study through semi-structured interviews. The participants were identified through professional networks, industry associations, and relevant government agencies. Participants with different experience levels, roles, and company sizes were included to enhance diversity.

In the Czech Republic, the survey included major entities representing approximately 60% of forest area ownership, such as the state enterprise Lesy ČR and military forests managed by VLS (Vojenské lesy a statky). In Slovakia, the survey focused on state forest areas managed by the state enterprise Lesy SR and other significant forest owners, covering more than 50% of forest land ownership. The panel also featured the second-largest game processor and the leading game product manufacturer in the Czech Republic, alongside the largest game meat processor in Slovakia, complemented by other representative processors. This diverse selection ensured that the data reflected a broad spectrum of industry practices and challenges. Respondents were anonymised using capital letters and numerals to maintain the confidentiality of commercially sensitive information, as shown in Table 1.

The interviews were conducted either face-to-face or via video conferencing platforms, depending on the availability and preference of the participants. Each interview lasted between 90 to 120 min, allowing for in-depth discussions on the selected topics.

### 2.3. Interview Structure

The interviews were structured around three main thematic areas designed to address the core research objectives of the study:

1. Consumer Preferences and Market Demand:

This theme explored consumer behaviour, preferences, and market trends for game meat in Slovakia and the Czech Republic. Questions focused on current demand, shifts in consumer interest, and key market segments, providing insights into consumer perceptions and opportunities for growth.

2. Market Growth and Value Chain Optimisation:

This area examined factors driving market growth, including production scalability, diversification of products, and supply chain improvements. Stakeholders were asked about infrastructure needs, strategies for market expansion, and addressing bottlenecks in distribution and processing.

3. Constraints, Barriers, and Competitiveness:

This theme combined production and regulatory challenges, focusing on legislative, technical, economic, and natural constraints. Stakeholders provided perspectives on regulatory barriers, supply chain inefficiencies, and socio-economic challenges. Discussions also covered competitiveness, pricing, and the strategic positioning of game meat relative to other meat industries.

### 2.4. Data Processing

The data processing phase began with the transcription of interviews to accurately capture qualitative data for analysis. The transcribed data were systematically coded into meaningful themes and sub-themes, enabling the identification of key patterns related to game meat production and marketing.

Thematic analysis was conducted to uncover recurring themes, focusing on production processes, marketing strategies, challenges, and opportunities. Comparative analysis highlighted similarities and differences across stakeholder groups and between Slovakia and Czechia, providing insights into varied approaches to game meat production and marketing.

Responses from stakeholders, categorized as primary producers, small processors, and large processors, were summarized and compared across three overview tables, corresponding to the three main thematic areas outlined in Section 2.3.

Data triangulation was employed by integrating primary interview data with secondary sources to ensure robust findings. This cross-verification from multiple perspectives enhanced the reliability and validity of the results, offering a comprehensive understanding of the game meat production and marketing landscape.

### 2.5. Analysis Procedures and Synthesis

The analysis included both descriptive and interpretive elements. Descriptive analysis detailed game meat production and marketing practices based on interview data, while interpretive analysis examined the meanings and implications of the identified themes within the broader context of game meat market dynamics. This dual approach combined factual reporting with contextual understanding.

Comparative analysis was performed at two levels: intra-country, to identify unique strategies, challenges, and market conditions in Slovakia and the Czech Republic, and inter-country, to highlight differences and similarities between the two nations. This provided a comprehensive view of regional dynamics shaping game meat production and marketing.

The findings from qualitative analysis and secondary data were synthesized to create a holistic understanding of the game meat industry in both countries. This synthesis integrated insights from multiple sources, offering a cohesive account of the industry’s complexity and broader implications.

## 3. Results

### 3.1. Characteristics of the Czech Republic and Slovakia in the Game Management Context

The Czech Republic and Slovakia exhibit distinct land-use patterns for hunting. In Slovakia, the total hunting area spans 4,446,273 hectares, with agricultural land comprising 52% (approximately 2.3 million hectares) and forest land accounting for 45% (roughly 2 million hectares) [40,41]. Conversely, the Czech Republic has a larger proportion of agricultural land, covering 57% (3.9 million hectares) of the total hunting area of 6,891,273 hectares, while forest land accounts for 38% (approximately 2.5 million hectares) (Table 2). These differences in land use directly influence the distribution and density of game species in each country.

**Figure 1:**
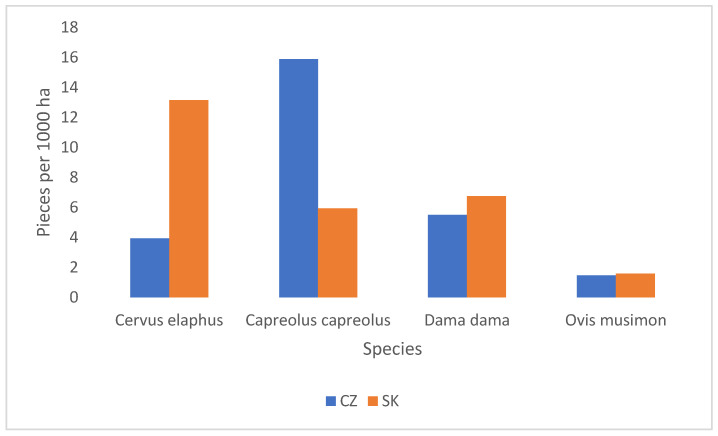
Number of main hunted ungulates per 1000 ha of hunting area.

Game species populations differ notably between the two countries, reflecting the respective land-use patterns. Slovakia sustains a higher population of red deer, with 58,518 hunted in 2022, compared to 32,884 in the Czech Republic. In contrast, the Czech Republic has significantly higher densities of wild boar (177,877 hunted) and roe deer (114,100 hunted), compared to Slovakia’s 52,163 wild boar and 26,392 roe deer (Table 3). These differences are influenced by Slovakia’s greater forest coverage, which favours red deer, while the Czech Republic’s reliance on agricultural land facilitates species like wild boar and roe deer (Figure 1).

Quantifying game-related damages remains a challenge in both countries. In 2022, reported damages were EUR 2.1 million in the Czech Republic and EUR 2.9 million in Slovakia, though these figures are considered underestimates due to incomplete reporting, primarily linked to damage claims [43,44]. Despite the limitations of the available data, increasing trends in reported damages highlight the growing ecological and economic impact of ungulate populations.

### 3.2. Qualitative Research: Key Findings

The thematic analysis of interviews revealed three major themes, each containing several sub-themes. The identified themes are the following: (1) consumer preferences and market demand, (2) market growth potential and value chain opportunities, and (3) constraints, barriers, and competitiveness. Within these broad themes, sub-themes such as price sensitivity, product diversification, branding, regulatory challenges, and seasonality of supply emerged as critical factors.

Each of the following sub-sections presents these themes in detail, first providing a narrative synthesis followed by a summary table of the responses obtained.

#### 3.2.1. Consumer Preferences and Market Demand

Czech landowners’ and users’ responses suggest a market with a niche interest in organic and healthy game meat but also marked hesitation from a significant portion of the respondents about the potential for increased production. Czech processors’ responses suggest a divergence in consumer preferences: some segments favour low-cost options, while others show interest in organic, healthy, or quality-driven products. This indicates potential opportunities for differentiation within the game meat market, depending on the target demographic.

Slovak landowners’ and users’ responses highlight a diverse market where some consumers prioritise affordability while others look for quality and brand recognition. Slovak processors perceive the demand in the Slovak game meat market as focused on quality, presenting an opportunity for premium products such as branded salami and other processed meats. However, there is also a price-conscious segment that values a balance between cost and quality.

#### 3.2.2. Market Growth and Value Chain Optimisation

Landowners and users in the Czech Republic identified health aspects, distribution channels, and branding as key factors for growing the game meat market. Three respondents saw potential in premium, value-added products, while one respondent focused on commoditization. Internet marketing was widely used to support sales, with some adding PR campaigns and articles. However, there was a gap in data usage, as only some respondents relied on consumer surveys and data analysis, while others had no access to market data, indicating missing data for informed decision-making.

Processors in the Czech Republic identified distribution channels, publicity, and price as the main factors for growing the game meat market. Most processors focused on improving processing efficiency and producing premium, value-added products to increase production. Internet marketing was a common tool for supporting sales, with some also using PR campaigns.

In Slovakia, landowners and users see consumer price, local brand development, and quality certification as key factors driving the growth of the game meat market. Opportunities for increasing production are largely focused on local communities, seasonality solutions, and quality certification. While most respondents use word of mouth or PR campaigns to support sales, no data or market analysis are currently being utilized, indicating a significant gap in strategic market insights.

Processors in Slovakia identified consumer education, quality of supply, and subsidized processing facilities as the primary drivers for game meat market growth. Opportunities to increase production focus on high value-added products and strict control of hunting quotas. Sales are primarily supported through shopper marketing, online channels, and word of mouth, though there is a lack of reliance on market data or analysis, indicating a gap in strategic decision-making tools.

#### 3.2.3. Production Constraints, Regulations, and Competitiveness

In the Czech Republic, landowners and users identified technical and legislative constraints as key limitations on game meat production, with three respondents citing these issues, while two respondents reported no such barriers. Game-in-hide buy-in prices were generally considered low, although two respondents noted a slight increase this year. The main obstacles to increasing production volume for retail consumers included distribution challenges for two respondents, while one respondent pointed to limited processing capacity, and one respondent highlighted low-profit margins as a major barrier to scalability.

The responses of Czech processors highlight the regulatory and logistical challenges faced by game meat processors in the Czech Republic, including legislative difficulties, inconsistent supply, and difficulties in retail distribution.

In Slovakia, landowners and users identified several production constraints in game meat production, with technical limitations such as hygiene issues, a lack of hunters, and weak distribution systems being the most common obstacles. Only one respondent did not report significant limitations. Game-in-hide buy-in prices were generally viewed as fair to low, with one respondent noting extremely low prices. The largest obstacles to increasing production volumes included strict regulations, seasonality of production, and consumer-related challenges, such as prejudices, lack of consumer experience, and health risk concerns, highlighting both supply chain issues and market hesitancy.

Slovak game meat processors identified several production constraints, including the low motivation of hunters, technical issues related to hygiene, retail prices competing with substitutes, and weak distribution during the off-season. Game-in-hide buy-in prices were mostly seen as fair, except for one processor, which reported extremely low prices, indicating a disparity in pricing perceptions across different processors. These constraints highlight both supply-side challenges and pricing pressures within the game meat sector in Slovakia.

### 3.3. Summary of Other Key Findings

Based on semi-structured interviews, additional data, including contextual and stakeholder insights, were identified alongside the thematic areas presented in Table 4, Table 5 and Table 6. These findings are synthesized in Table 7, organized by country and specific roles within the value chain.

## 4. Discussion

This study unveils dynamics in game meat production and marketing in Slovakia and the Czech Republic. The results, as summarized in Table 4, Table 5 and Table 6, provide valuable insights into consumer preferences, market growth opportunities, and production constraints.

**Market Demand and Consumer Preferences (Table 4)**
  Stakeholder responses from Table 4 indicate that consumer demand varies to some extent between Slovakia and the Czech Republic. In Slovakia, consumers show a stronger focus on affordability, while in the Czech Republic, premium and branded products are more in demand. This difference aligns with broader trends in consumer purchasing behaviour influenced by income levels, brand awareness, and media exposure. Furthermore, organic and sustainable game meat products are emerging as a niche market in both countries. However, challenges such as price sensitivity and limited branding efforts hinder market expansion in Slovakia.

**Market Growth and Value Chain Optimisation Opportunities (Table 5)**

As highlighted in Table 5, stakeholders identified opportunities for value chain optimisation through improved processing infrastructure, distribution networks, and product diversification. Health-related perceptions and rising beef and pork prices present opportunities to position game meat as a competitive alternative. Additionally, localized processing and branding initiatives could enhance market penetration in Slovakia. In both countries, strategies such as shortening distribution chains and subsidizing processing facilities for off-season production can improve market accessibility.

Recent advancements in supply chain management, particularly the integration of circular economy principles, offer further opportunities for improving sustainability and efficiency in the game meat value chain. Research on green centralized supply chains highlights strategies for reducing waste, streamlining operations, and minimizing environmental impacts [45]. In the context of the game meat sector, centralized processing hubs could address inefficiencies caused by seasonality, as these facilities would enable better coordination across stakeholders, reducing downtime during off-peak periods.

Moreover, contractual models such as revenue-sharing agreements and quantity discounts could align incentives among hunters, processors, and distributors, fostering greater collaboration and risk-sharing across the value chain [45]. These strategies would encourage small-scale producers to participate in formalized networks, reduce reliance on intermediaries, and improve profitability. Additionally, incorporating circular economy approaches—such as recovering and reusing by-products like bones and hides for secondary purposes (e.g., fertilizers or animal feed)—can generate secondary revenue streams, supporting economic sustainability while reducing waste.

**Production Constraints and Competitiveness (Table 6)**

Table 6 emphasizes the role of legislative and technical barriers, seasonality of supply, and market hesitancy in limiting production volumes. In Slovakia, strict regulations, low hunter motivation, and challenges in meeting hygiene standards constrain production. Similarly, Czech processors face distribution challenges and pricing pressures from retail chains. Addressing these barriers requires tailored policy interventions, enhanced collaboration among stakeholders, and investments in processing infrastructure.

Building on recent studies, addressing these constraints through digital transformation and demand prediction tools could help producers and processors manage the unpredictability of seasonal supply [45]. For example, GIS mapping, thermal imaging, and demand forecasting models could be leveraged to improve real-time decision-making and reduce waste. These technologies would enhance supply chain visibility and resilience, particularly during peak hunting seasons, when demand often exceeds processing capacity.

Together, these findings underline the need for strategic interventions to address demand–supply mismatches, optimise value chains, and overcome production challenges to unlock the full potential of the game meat market.

### 4.1. Challenges in Game Management: Slovakia vs. Czech Republic

As it is evident from Section 3.1, Characteristics of the Czech Republic and Slovakia in the Game Management Context, the ecological and economic contexts of Slovakia and the Czech Republic shape distinct challenges in game management. As apparent from the Table 2, Slovakia’s extensive forest coverage supports large red deer populations, necessitating stricter quotas and causing seasonal bottlenecks in processing capacity. In contrast, the Czech Republic’s agricultural landscape fosters higher densities of wild boar and roe deer, creating year-round demand but also escalating crop damage and population growth issues. Economically, Slovak producers face intense price competition from conventional meats, while Czech processors contend with cross-border competition within the EU. Regulatory frameworks, though harmonised with EU standards, disproportionately burden small producers in both countries, fragmenting the market and hindering growth.

Hunters, as primary producers in the game meat supply chain, play a crucial role in supplying raw meat to processors. However, their declining motivation—driven by mandatory hunting quotas, inadequate economic incentives, and regulatory challenges—negatively impacts the entire value chain. As shown in Table 6, low purchase prices fail to compensate for the high workload involved in game management, forcing hunters to sell whole carcasses at low margins to processors. Regulatory requirements, such as strict hygiene standards, limit hunters’ ability to produce value-added products, further reinforcing dependence on intermediaries.

This dependency fosters grey-market practices, with some hunters bypassing formal channels to achieve higher returns. Such practices compromise traceability, create unfair competition, and erode consumer trust. Additionally, the substantial capital investment required for small-scale processing facilities restricts the commercialisation of pre-sliced game meat, locking producers into low-value sales and increasing reliance on large processors, as indicated in Table 5 and Table 7.

Game meat production in both countries operates under a tiered regulatory framework designed to ensure food safety and align with European Union (EU) standards [46]. Producers are required to meet varying levels of compliance based on their scale of operation as indicated in Table 6:

- **Local Distribution**: Minimal regulatory requirements allow for the sale of unprocessed game meat directly to consumers in its natural state (e.g., in fur or feathers).
- **Small-Scale Processing**: Moderate regulations govern small facilities, limiting the volume of processed game meat and necessitating investments in basic infrastructure.
- **Large-Scale Processing**: The most stringent tier applies to industrial facilities, requiring significant financial and administrative commitments to ensure premium quality and safety standards.

While these regulations aim to harmonize safety practices, they disproportionately burden smaller producers who lack the resources to meet compliance demands. Streamlined licensing processes, subsidies for infrastructure, and tailored support could reduce barriers and promote broader participation in the market.

Inefficiencies in the distribution chain exacerbate these challenges. Lengthy and fragmented networks concentrate profits within retail chains, leaving producers and processors with minimal returns. Urban consumers, accustomed to convenient purchasing options, face limited access to facilities capable of processing in-fur carcasses, further constraining demand. This trend reflects broader lifestyle shifts, where traditional game meat consumption struggles to maintain relevance.

The fragmented supply chain further complicates the industry, as shown in Table 6, leading to inefficiencies in processing, distribution, and product availability. Key issues include the following:

- **Infrastructure Gaps**: Insufficient processing capacity during peak hunting seasons leads to delays, increased costs, and reliance on low-value raw sales. In Slovakia, the red deer hunting season creates concentrated demand, leaving facilities underutilized for much of the year.
- **Coordination Issues**: Limited collaboration among stakeholders, including producers, processors, and distributors, results in higher operational costs and inconsistent product quality.
- **Traceability**: Effective tracking systems, essential for food safety, require substantial investment, posing a challenge for smaller producers.

Seasonal fluctuations exacerbate these issues, making it difficult to maintain consistent supply and meet market demand year-round. Addressing these inefficiencies is essential to enhance the sector’s resilience and competitiveness.

To synthesise the challenges and opportunities presented, a SWOT analysis of the game meat sector offers a concise overview of its current positioning in Slovakia and the Czech Republic.

**A SWOT analysis found the following:**
**Strengths:**

- Growing consumer preference for organic and sustainable food products;
- Strong cultural and historical significance of game meat in both Slovakia and the Czech Republic;
- Established hunting traditions and regulatory frameworks supporting production;
- Diverse ecosystems fostering a variety of game species.

**Weaknesses:**

- Inconsistent supply chains and seasonal production cycles impacting market stability;
- High processing costs and regulatory burdens limiting market entry for smaller producers;
- Limited consumer awareness and accessibility in urban areas;
- Dependence on traditional distribution channels, which restricts scalability.

**Opportunities:**

- Expansion into premium and export markets, particularly in Western Europe;
- Development of value-added products like ready-to-cook meals, premium sausages, and preserves;
- Leveraging e-commerce and digital marketing to enhance accessibility and consumer engagement;
- Collaboration between stakeholders to establish shared processing infrastructure and streamline supply chains.

**Threats:**

- Competition from other organic meat sources, such as poultry and beef;
- Regulatory challenges, particularly EU veterinary and hygiene standards, which disproportionately affect smaller producers;
- Vulnerability to changes in hunting quotas and environmental regulations;
- Economic downturns potentially reducing consumer spending on premium products;
- Public misconceptions about hunting ethics and sustainability, which may impact market perception.

### 4.2. Ecological Considerations and Sustainable Wildlife Management

Game management plays an important role in maintaining ecological balance, especially in regions with high densities of ungulates [47,48,49]. Both Slovakia and the Czech Republic face ecosystem degradation linked to overpopulation of species like red deer, roe deer, and wild boar. This overabundance leads to extensive browsing pressure, damaging forest regeneration, reducing biodiversity, and degrading soil health. It also exacerbates human–wildlife conflicts, particularly in agricultural zones, where crop damage reduces yields and profitability [6].

To address these ecological impacts, a more adaptive approach to game management can be considered:

- **Dynamic Quota Systems:** Reliable implementation of flexible quotas based on real-time ecological monitoring and habitat assessments can optimise population control while aligning harvest levels with market demand. Using drone surveys, thermal imaging, and GIS mapping can improve accuracy in estimating population densities and habitat conditions, allowing for evidence-based decisions.
- **Landscape Restoration and Habitat Management:** Complementing hunting efforts with consistent and monitored habitat restoration—such as reforestation, fencing of sensitive regeneration areas, and planting forage species in buffer zones—can reduce browsing damage and improve biodiversity resilience.
- **Integrated Damage Mitigation Strategies:** Developing compensation schemes for agricultural losses and incentivizing farmers to collaborate in wildlife-friendly land management can reduce tensions between conservation goals and agricultural interests. These approaches could also include non-lethal deterrents, such as scent barriers or fencing, in high-risk areas [50].
- **Promoting Ecosystem Services:** Highlighting the ecological role of hunting in biodiversity conservation and habitat protection can enhance public perception and support for regulated game meat production. This requires public education campaigns and partnerships with environmental organizations to shift the narrative from “hunting as sport” to “hunting as ecological stewardship”.

### 4.3. Addressing the Research Questions

**Research Question 1 was the following:** What influences consumer preferences and purchasing behaviour toward game meat, and how do these differ between Slovakia and the Czech Republic?

Consumer preferences in Slovakia and the Czech Republic are shaped by several key factors, as evidenced by Table 4 and Table 5, and the qualitative findings are presented in Section 3.2.1 and Section 3.2.2:

- Health Perceptions: Game meat is valued for its “organic”, “sustainable”, and “low-fat” qualities.
- Price Sensitivity: Slovak consumers are particularly price-conscious, often comparing game meat to conventional proteins like poultry, pork, and beef.
- Cultural Acceptance and Culinary Exposure: Marketing campaigns, cooking shows, and educational efforts enhance the acceptance and familiarity with game meat.

In the Czech Republic, the consumer base is diverse, with segments prioritizing affordability and others seeking premium, health-oriented products with regional branding. In Slovakia, cost competitiveness is a dominant factor, but an emerging segment shows interest in higher-value branded products. Targeted communication strategies, clear labelling of health and ecological attributes, and media promotion can expand consumer acceptance in both countries.

**Research Question 2 was the following:** What value chain improvements can optimise game meat market growth?

Research highlights several opportunities to strengthen and grow the game meat sector, supported by findings from stakeholder interviews and data analysis presented in Table 4, Table 5 and Table 6:

- Value-Added Products: Developing branded, high-quality products like specialty sausages, salamis, and ready-to-cook kits can move the sector beyond price-based competition (Table 5). The responses emphasized growing interest in organic and high-quality game meat products, driven by cooking shows and health trends (Section 3.2.1).
- Shared Processing Infrastructure: Cooperatives or shared facilities can help small producers reduce costs and improve processing consistency (Table 5).
- Direct Sales and E-Commerce: Online platforms for in-fur carcasses and processed cuts provide opportunities to bypass retail chains and secure better margins (Table 5 and Table 6).
- Consumer Education: Educational campaigns on preparing and cooking game meat, aligned with sustainability narratives, can boost demand, especially among premium market segments (Table 5).
- Seasonality Solutions: Mobile processing units, short-term storage, and flexible staffing can address supply fluctuations caused by seasonal hunting peaks (Table 5 and Table 6).

**Research Question 3 was the following:** What factors impact game meat production, profitability, and market access, and what reforms are needed to address these challenges?

Game meat production in Slovakia and the Czech Republic faces several challenges, including regulatory, technical, economic, and natural constraints (Table 6 and Table 7):

- Regulatory Burdens: EU veterinary and hygiene standards can be costly and complex, particularly for small producers, exacerbated by varying regional interpretations.
- Infrastructure Limitations: Seasonal hunting creates bottlenecks in processing capacity, especially for red deer in Slovakia. The Czech Republic benefits from more consistent wild boar harvesting.
- Economic Pressures: Low buy-in prices discourage hunters from meeting quotas and investing in higher-quality processing. Slovak producers face price competition from conventional meats, while Czech producers contend with cross-border competition.
- Natural Constraints: High ungulate densities lead to forest damage, requiring regulated culling, but stringent quotas can create social tensions.
- Slovakia should focus on adaptive quotas and streamlined processing during peak hunting seasons to manage red deer populations effectively. Meanwhile, the Czech Republic requires continuous management strategies for wild boar and roe deer to mitigate agricultural damage and support sustainable hunting practices.

### 4.4. Strategic Reforms to Strengthen the Sector

Building on the SWOT analysis, research findings, and identified challenges, and addressing the research questions on consumer behaviour, value chain optimisation, and regulatory barriers, the following measures and reforms are proposed to enhance regulatory frameworks, improve infrastructure, and foster collaboration within the sector, ensuring long-term sustainability and growth:

- **Simplifying Compliance**: Scaled licensing and inspection requirements for small producers can reduce costs while maintaining safety standards. Reducing administrative complexity can encourage broader participation, particularly from small-scale producers.
- **Infrastructure Investment**: Subsidies for cold storage, mobile processing facilities, and cooperative plants can support distributed production models. Enhanced infrastructure would enable consistent supply chains and reduce seasonal bottlenecks.
- **Flexible Quotas**: Dynamic quota systems based on real-time surveys can balance ecological needs with economic realities. This approach allows an adaptive management that considers both market demand and biodiversity conservation.
- **Market Oversight**: Improved tracking systems and actions can reduce illegal market activities, along with affordable options for small producers. Stronger oversight builds trust with consumers and ensures fair competition among producers.
- **Consumer Education**: Public campaigns can highlight the ecological benefits of game meat consumption and sustainable hunting practices. Education can improve market perception and expand the consumer base by addressing misconceptions about hunting ethics.

### 4.5. Study Limitations

While this study provides insights into the game meat sector in Slovakia and the Czech Republic, which we consider deeply representative, several limitations should be acknowledged.

First, the reliance on qualitative interviews introduces the possibility of respondent bias, as participants may have subjective perceptions influenced by their personal experiences and roles in the supply chain. Although efforts were made to ensure diverse representation, the findings may not capture the full spectrum of views within the industry.

Second, the regional focus on Slovakia and the Czech Republic limits the generalizability of the results to other contexts. Game meat production and marketing dynamics may vary significantly across countries, influenced by cultural, ecological, and regulatory differences. Future research could expand the geographical scope to include other European nations for comparative analysis to extend our finding within this wider context.

Third, the study’s emphasis on qualitative data limits the ability to quantify the magnitude of the challenges and opportunities identified. Integrating quantitative approaches, such as focused market surveys, could provide additional insights and strengthen the quantitative robustness of the findings.

## 5. Conclusions

The findings from Slovakia and the Czech Republic provide valuable insights into the global game meat industry, emphasising the need for integrated supply chains, innovative processing solutions, and targeted consumer education. These strategies are essential for balancing ecological sustainability with economic viability in wildlife management.

Game meat represents a unique opportunity to connect ecological management with sustainable food production while contributing to rural development. It fosters employment, supports local economies, and preserves cultural heritage. Synergies with rural tourism and gastronomy—such as farm-to-table events, culinary festivals, and hunting tours—can enhance the sector’s value and strengthen producer–consumer relationships [9,46]. Agri-environmental subsidies promoting sustainable hunting and processing practices can further incentivise environmentally and economically viable approaches [51,52].

Government support is pivotal in addressing structural challenges. Investments in infrastructure, reductions in regulatory burdens, and promotion of consumer awareness are critical enablers. Subsidies, cooperative models, and marketing campaigns are essential for stabilising supply chains and unlocking the industry’s potential.

By addressing current challenges and implementing innovative strategies, the game meat industry has the potential to expand globally, contributing to biodiversity conservation, rural development, and the premium food market. Sustained progress, however, will require targeted research in several critical areas:

- **Consumer Behaviour**: Investigating global consumer preferences and purchasing patterns to inform international marketing strategies and expand market reach;
- **Environmental Benefits**: Exploring the role of game meat production in biodiversity management and its broader ecological advantages;
- **Technological Advancements**: Assessing innovations like mobile processing units and traceability systems to improve efficiency, transparency, and market access;
- **Regulatory Best Practices**: Conducting comparative studies of regulatory frameworks to identify harmonised policies that enhance sustainability and competitiveness.

Game meat production strategies can also align with broader initiatives like the EU Green Deal [53] and the Farm-to-Fork Strategy [54] to promote sustainable development.

The following actions are recommended to enhance the EU policy relevance to the study:

- **Introduce Financial Incentives**: Establish targeted Common Agricultural Policy subsidies for organic game certification, sustainable hunting, and ethical processing methods to promote sustainability;
- **Strengthen Digital Tracking Technology**: Expand the use of digital marketing technology to improve food safety, market confidence, and transparency. Establishing uniform EU-wide standards for wild game meat certification could facilitate trade and consumer trust.
- **Reduce the Carbon Footprint**: Invest in low-emission processing technologies, sustainable packaging, and short-chain logistics to decrease the environmental impact of game meat production.
- **Prioritise Circular Economy Principles**: Focus on waste minimisation, by-product utilisation, and renewable energy adoption in processing to align with sustainability goals.
- **Promote Sustainable Diets and Consumer Awareness**: Launch public information campaigns to highlight the environmental and nutritional benefits of game meat, integrating these efforts into EU Farm-to-Fork Strategy initiatives, such as school food programs and sustainable procurement policies.
- **Support Rural Development and Biodiversity Conservation**: Encourage community-led cooperative models that integrate game meat production with biodiversity-friendly practices, supported through EU Rural Development Programs.

Through a coordinated effort combining innovation, policy reform, and stakeholder engagement, the game meat industry can achieve its full potential as a model for sustainable food production and ecological stewardship.

## Figures and Tables

**Table 1:** CZ and SK respondents’ structure.

Country	Role	Number	Codes
The Czech Republic	Land owners and users *	5	CL1, CL2, CL3, CL4, CL5
	Processors *	7	CP1, CP2, CP3, CP4, CP5, CP6, CP7
Slovakia	Land owners and users *	6	SL1, SL2, SL3, SL4, SL5, SL6
	Processors *	4	SP1, SP2, SP3, SP4

* Some landowners are actively involved in game meat processing, while certain processors also hold positions within the governing bodies of hunting associations. Respondents were categorized based on their primary activities, but those with dual roles provided insights from both perspectives.

**Table 2:** Comparative overview of the Czech Republic and Slovakia [40,41,42].

Characteristic	Czech Republic (Year)	Slovakia (Year)
Total forest area (ha)	2,680,372 (2022)	2,027,481 (2022)
Forest cover (%)	34.2% (2022)	41.4% (2022)
Ownership structure	71.79% Public, 28.21% Private	58.03% Public, 41.97% Private
Main tree species composition	Spruce (46.8%), pine (16%), beech (9%), oak (7.8%) (2022)	Beech (35%), spruce (21.3%), oak (10.3%), pine (6.5%) (2022)
Hunting area (ha)	6,891,273 (2022)	4,446,273 (2022)
Type of hunting land	Field—57%, Forest—38%, and Other—5% (2022)	Field—52%, Forest—45%, and Other—3% (2022)

**Table 3:** Number of main hunted ungulates (pcs) in CZ and SK (2022).

Species	Czech Republic	Slovakia (2022)
Roe Deer (*Capreolus capreolus*)	114,100	26,392
Red Deer (*Cervus elaphus*)	32,884	58,518
Fallow Deer (*Dama dama*)	38,653	30,082
Mouflon (*Ovis musimon*)	10,245	7059
Wild Boar (*Sus scrofa*)	177,877	52,163

**Table 4:** Responses to the thematic area—Market Demand and Consumer Preferences.

	CZ	SK
**Do you perceive a demand for an increase in game meat production?**		
**Landowners and users**	A little (2), No (3)	A little (2), Yes (3), No (2)
**Processors**	A little (4), Yes (2), Rather decrease on the consumer market	Yes (3), A little
**What types of game meat products are in demand? What are the current trends?**		
**Landowners and users**	Organic, healthy meat (3), TV cooking shows (2), No answer	Price/quality (3), Quality, brands, Low cost
**Processors**	Cooking shows trends, Low cost (2), Driven by price, Organic food, healthy food, Quality Price/quality balance	High quality/salami, etc./, Brands (2), Price/quality (2), Quality

**Table 5:** Responses to the thematic area—Market Growth Potential and Value Chain Opportunities.

	CZ	SK
**What are the factors that could contribute to the potential growth of the game meat market?**		
**Landowners and users**	Health aspects (3), distribution channels (2), social trending, publicity, Brand	Consumer education (2), Consumer price, Local brands development, Quality and continuity of local supply, Rising prices of beef and pork, Quality certification, Health and accessibility promotion
**Processors**	Price (2), Distribution channels (2), Logistics/availability of inputs (2), publicity/campaigns/ (2), Health aspects, Quality standards	Consumer education, rewarding compensation for hunters, Quality and continuity of supplies, Rising prices of beef and pork, shortening the distribution chain, Subsidized processing facilities for the off-season, supply to schools and government facilities
**Where do you perceive the possibility of increasing game meat production?**		
**Landowners and users**	Premium products with higher value-added (3), New product development, premium products, improvement of processing efficiency, Commoditization	Local communities, Seasonality solution, hunting quotas verification, Processing capacity increase for the season period, Hunting quotas verification, local processing, Origin and quality certification, hyperlocal economy, Local community support, final products locally processed
**Processors**	Increase in processing capacity and retail distribution channel coverage, Improvement of processing efficiency (waste logistics) (2), Premium products with higher value-added (2), distribution channels coverage (retail), logistics (interim storages for minimum amount transportation), Quality standards introduction (freshness, lead content, protein content) (2)	Processing products with high value-added, Local processing capacity, hunting quotas verification, Hunting quotas verification, local processing, Hunting quotas adherence, strict control, hunters’ subsidies, buy-in guaranteed price
**What steps are you taking to support the sale of game meat?**		
**Landowners and users**	Internet marketing (5), PR campaigns, pick-up points (2), PR articles	Word of mouth reputation (2), No answer, PR articles, brand, High quality, hunting tourism, Hunting society promotion amongst members
**Processors**	Direct marketing, internet marketing, Direct marketing, internet marketing (5), Direct marketing (2), PR articles (2), sales representative/commission-based	Shopper marketing, brand, E-shop, B2B long-term relationships, Word of mouth reputation, quality control, Sample retail shops, brand, PR articles
**Do you have any data (market analyses, marketing, etc.) you use?**		
**Landowners and users**	Consumer surveys (3), demographic data analysis, internal economic data analysis, articles research, No answer, No	No (6)
**Processors**	Consumer surveys (5), internal sales data (2), regular meetings with key clients, statistical and demographic data analysis, No	No answer (2), No (2)

**Table 6:** Responses to the thematic area—Production Constraints and Competitiveness.

	CZ	SK
**Are there existing limitations on game meat production (legislative, technical, and/or natural)?**		
**Landowners and users**	Legislative (3), technical (2), No (2),	Technical (3), lack of hunters (3), Missing processing capacity, weak distribution, Hunting offseason, hygiene regulation, retail prices, No
**Processors**	No (3), legislative (unequal processing conditions) (3), Market quality standards,	Low motivation of hunters, Technical (hygiene for processing), Retail prices versus substitutes, Off-season weak distribution
**What is the largest obstacle to increasing the production volume for retail** **consumers?**		
**Landowners and users**	Distribution channels to retail (2), Limited processing capacity, Low-profit margin, No answer	Price (3), too strict regulation, Seasonality of production, Prejudices, conservativism, Lack of consumer experience, Retail prices, health risk worries, consumer package unavailability
**Processors**	Distribution channels to retail (3), Low quantities suitable for processing (2) Irregularity of supply, Qualified personnel	Price (2), Lack of game meat supplies, especially off-season, Prejudices, conservatism, Lack of consumer experience, variable quality

**Table 7:** Summary of key players’ semi-structured interview outcomes.

Value Chain Role	CZ	SK
**Primary producers**	Without significant investment, it is impossible to market and sell sliced meat; only whole carcasses with in-fur can be distributed. State subsidies for refrigeration equipment have been beneficial. The purchase prices from large-scale buyers have been stagnating for an extended period. In the “grey” market (in violation of regulations), some individuals offer meat portioning services.	Limited consumer demand for in-fur carcasses. Extremely low industrial buying prices. Strict hunting quotas (high) compared to demotivated hunters (only 25% of registered hunters are performing active hunting). Some individuals offer meat portioning services in the grey market (in direct violation or outsmarting of regulations). Some hunting societies purposely do not meet minimum hunting quotas which puts a burden on their neighbours due to the game migration.
**Small processors**	Highly complex veterinary regulations. Complex and demanding technology. 67% of the produced product can only be sold at the butchering location, and 33% can be sold to restaurants and retail stores, which leads to problematic distribution.	Financially impossible to enter retail chains with basic-cut meat. The best experience is farm gate sales with word-of-mouth quality references. Prices must be comparable to regular meat in retail for the basic offer.
**Large processors**	Continuous veterinary supervision is costly. Official processors must pay for waste disposal. Hunters illegally skin game (and sell sliced meat), whereas waste is left in the forest. Extreme price pressure when selling to retail chains. In border regions, there is competition from buyers in other EU countries. There is a lack of qualified butchery personnel.	Seasonal game meat supply, significant competition for procurement outside the main hunting season. With high price pressure when selling to retail chains, some open their proprietary retail stores. Challenging to retain meat processing plant employees outside the main season (relocation to other activities). Prices for large purchases are heavily influenced by global New Zealand supply.

## Data Availability

Data is available upon e-mail request made to the corresponding author.

## Abbreviations

The following abbreviations are used in this manuscript:

CSO	Czech Statistical Office
MPRS	Ministry of Agriculture and Rural Development of the Slovak Republic
NLC	National Forest Centre
SPK	Slovak Hunting Chamber
UHUL	The Forest Management Institute Brandýs nad Labem
VLS	Vojenské lesy a statky

## Appendix A

Areas of qualitative interview with focused subsections included the following:

1. Consumer Preferences and Market Demand:
  - Do you perceive a demand for an increase in game meat production for Czech/Slovak consumers?
  - What types of game meat products are in demand? What are the current trends?
  - Where is the main market for the game meat you produce?
2. Market Growth Potential and Value Chain Opportunities:
  - What are the factors that could contribute to the potential growth of the game meat market?
  - Where do you perceive the possibility of increasing game meat production?
  - What steps are you taking to support the sale of game meat?
  - What are the other stages of the supply chain?
3. Constraints, Barriers, and Competitiveness:
  - Are there existing limitations on game meat production (legislative, technical, and/or natural)?
  - What is your perception of current game-in-hide buy-in prices?
  - What is the largest obstacle to a production volume increase to retail consumers?
  - Who do you consider as competitors from the product (game meat) competitive market perspective?
  - What is the flexibility in changing production (e.g., from frozen to semi-finished products, etc.)?
  - In your view, what are the key challenges and barriers currently affecting the game meat production sector, and how do you believe these challenges could be addressed to ensure a sustainable future for the industry?
